# Worldwide Heterogeneity of Food Allergy: Focus on Peach Allergy in Southern Italy

**DOI:** 10.3390/jcm13113259

**Published:** 2024-05-31

**Authors:** Valentina D’Aiuto, Ilaria Mormile, Francescopaolo Granata, Filomena Napolitano, Laura Lamagna, Francesca Della Casa, Amato de Paulis, Francesca Wanda Rossi

**Affiliations:** 1 Department of Translational Medical Sciences, University of Naples Federico II, 80131 Naples, Italy; valentina.daiuto@unina.it (V.D.); ilariamormile87@gmail.com (I.M.); filomena.napolitano@unina.it (F.N.); francescadellacasa4@gmail.com (F.D.C.); depaulis@unina.it (A.d.P.); francescawanda.rossi@unina.it (F.W.R.); 2 Department of Clinical Medicine and Surgery, University of Naples Federico II, 80131 Naples, Italy; lauralamagna1@gmail.com; 3 Center for Basic and Clinical Immunology Research (CISI), WAO Center of Excellence, University of Naples Federico II, 80131 Naples, Italy

**Keywords:** allergy, epidemiology, component-resolved diagnostics, allergens, food allergy, lipid transfer proteins, Pru p 3

## Abstract

Food allergy (FA) has shown an increasing prevalence in the last decades, becoming a major public health problem. However, data on the prevalence of FA across the world are heterogeneous because they are influenced by several factors. Among IgE-mediated FA, an important role is played by FA related to plant-derived food which can result from the sensitization to a single protein (specific FA) or to homologous proteins present in different foods (cross-reactive FA) including non-specific lipid transfer proteins (nsLTPs), profilins, and pathogenesis-related class 10 (PR-10). In addition, the clinical presentation of FA is widely heterogeneous ranging from mild symptoms to severe reactions up to anaphylaxis, most frequently associated with nsLTP-related FA (LTP syndrome). Considering the potential life-threatening nature of nsLTP-related FA, the patient’s geographical setting should always be taken into account; thereby, it is highly recommended to build a personalized approach for managing FA across the world in the precision medicine era. For this reason, in this review, we aim to provide an overview of the prevalence of nsLTP-mediated allergies in the Mediterranean area and to point out the potential reasons for the different geographical significance of LTP-driven allergies with a particular focus on the allergenic properties of food allergens and their cross reactivity.

## 1. Introduction

Food allergy (FA) is defined as an adverse reaction to a specific food antigen that would normally be harmless. In the last few decades, FAs have represented a common condition with an increasing prevalence and have become a major public health problem since it affects about 3–10% of children and up to 10% of adults [1]. However, data on the prevalence of FA across the world are heterogeneous because they are influenced by several factors [2]. Several mechanisms are causative of FA: immunoglobulin E (IgE)-mediated FAs, non-IgE-mediated, and mixed [3]. However, most data refer to the IgE-mediated FA because it is widely distributed worldwide, its pathogenetic mechanisms are better characterized, and its diagnostic tools are routinely available [4]. Among IgE-mediated FA, an important role is played by FA related to plant-derived food which can result from the sensitization to one protein of a single food (specific FA) or homologous proteins present in different foods with small differences between them (cross-reactive FA) [5,6]. Cross-reactive allergens in plant-derived foods include non-specific lipid transfer proteins (nsLTPs), profilins, and pathogenesis-related class 10 (PR-10). The clinical presentation of FA is widely heterogeneous ranging from mild symptoms to severe reactions up to anaphylaxis, most frequently associated with nsLTP-related FA (LTP syndrome). The purpose of our study is to describe the prevalence of food allergy in different world geographical areas by giving, in particular, an overview of the non-specific lipid transfer protein (LTP)-mediated allergies in the Mediterranean area and pointing out the potential reasons for the different geographical significances of LTP-driven allergies.

## 2. Epidemiology

FA affects people of all socioeconomic and demographic conditions affecting about 3–10% of children and up to 10% of adults [1,7]. The prevalence of FA has increased worldwide in the last 20 years, particularly in Westernized developed countries [8]. The estimation of the prevalence of FA is conditioned by lots of factors such as the intrinsic characteristics of individuals (i.e., age, gender, ethnicity, genetics), geographical setting (i.e., urban and metropolitan areas vs. rural ones, the presence of air pollution, different climatic zones with different vegetation and pollen distribution, various available foods), dietary habits or practices (i.e., usual foods, the custom of cooking foods and cooking method used, breastfeeding duration and age of weaning and introduction of solid food, dietary fat), socioeconomic and racial disparities (i.e., different awareness of food allergy, inequality of health care access and utilization, availability and use of drugs or diet supplementation) and the methodology used for diagnosis [9,10,11,12,13,14,15,16,17,18]. The high number of combinations of these factors in the various geographical areas justifies the wide heterogeneity of the worldwide prevalence of FA which ranges from a minimum of 0.14% reported in India [19] to a maximum of 37.8% reported in Europe [9,10,20].

Large double-blind, placebo-controlled food challenge studies (DBPCFC) and an oral food challenge (OFC) represent the gold standard for the diagnosis of FA [1,21]. However, they are expensive and associated with the risk of serious allergic reactions. Thus, they are often replaced by different markers for the determination of FA which include self-reported clinical history of FA, medical examination for the diagnosis of FA, performance of skin prick tests (SPTs), or measurement of allergen-specific IgE [1,21,22]. Recent European Academy of Allergy and Clinical Immunology (EAACI) guidelines provided recommendations for the diagnosis of IgE-mediated FA in the presence of a history focused on allergy and defined probable FA as the combination of typical clinical symptoms of FA together with IgE sensitization to the same food [1,23].

Two large population-based surveys, conducted in the United States, found that 7.6% of children [24] and 10.8% of adults [25] had probable FA. In the Australian population, the Melbourne Health Nuts and School Nuts studies show prevalence rates from 3.8% to 11% in infants [26,27] and to 4.5% in young adolescents [28]. In Europe, as part of the EuroPrevall project, screening questionnaires were administered to a random sample of adults in eight major European metropolitan regions. The authors estimated the prevalence of FA in three different conditions: (i) self-reported food allergy prevalence to any food; (ii) the prevalence of food allergy referring to one of the 24 foods considered priority foods (chicken eggs, cow’s milk, fish, shrimp, peanuts, hazelnuts, walnuts, peaches, apples, kiwi, melon, banana, tomato, celery, carrot, corn, lentils, soybeans, wheat, buckwheat, sesame seeds, seeds mustard, sunflower seeds, and poppy seeds); and (iii) the prevalence of probable FA in at least one of the above priority foods. A huge variability between countries in the prevalence of FA was observed, with rates of self-reported allergy to any food ranging from 1.7% to 37.3%, self-reported allergy rates to at least one priority food ranging from 0.5% to 18.9%, and rates of probable FA to at least one priority food ranging from 0.3% to 5.6% [20]. Extension of the same project in children showed that the prevalence of probable FA ranged from 1.9% to 5.6% [23]. In Italy, a study conducted on adult patients from seventeen allergy clinics scattered in different Italian areas revealed a prevalence of IgE-mediated FA of 8.5% [29]. In various countries from Asia, South and Central America, and Africa, reliable epidemiological data about FA are limited [21]. In Asia, the data from a multicenter epidemiological survey conducted on children recruited from China (Hong Kong and Guangzhou as metropolitan areas, and Shaoguan as a rural area), Russia, and India showed that the prevalence of probable FA was highest in Hong Kong (1.50%), intermediate in Russia (0.87%), and lowest in Guangzhou (0.21%), Shaoguan (0.69%), and India (0.14%) [19]. FA in South and Central America, and Africa is underdiagnosed and the data currently available are not sufficient to carry out systematic reviews, and the studies, involving a limited number of countries, are not representative of the vast and heterogeneous African context [30].

## 3. Pathogenesis

Food allergy is a pathological, potentially life-threatening immune reaction triggered by harmless food protein antigens [31]. FA is multifactorial and is the result of a complex interplay of genetic, dietary, and environmental factors [21]. The genetics of food allergy has been studied by several research groups. Results from the National Health and Nutrition Examination Survey 2005–2006 (NHANES) demonstrated that food allergy can vary with ethnicity showing a 4-times higher prevalence in African Americans than European Americans [32]. In particular, in the pediatric population, African American children present a significant increase in the prevalence of peanut allergy than the general United States pediatric population [32,33]. A possible explanation can be found in a higher prevalence of some variants of genes encoding for Th2-related molecules such as interleukin (IL)-4 and IL-13 in African Americans [34]. In addition, these subjects show increased levels of IgE, T helper 2 cytokines, and peripheral eosinophils as compared to European Americans [35,36]. However, some genetic variants only act as risk enhancers in case of environmental exposure to specific triggers [37]. Gene–environment interactions in allergy development have been evaluated in different allergic patient populations [38,39]. For example, exposure to air pollution in the presence of genetic risk factors is commonly associated with the development of bronchial asthma [40,41]. With regard to food allergy, most of the epidemiological differences observed are due to variations in the physical, social, and economic environment rather than in the genetic pool as demonstrated in studies on twins or emigrants presenting with marked differences as compared to their counterparts living in the native country [32,42,43,44]. The environmental factors possibly involved in this process are dietary habits, allergen exposure (including foods, allergens, and pets), pollution, vitamin D intake, hygiene-related factors, infections, and gut microbiome [45,46,47]. Another environmental factor is the time and the route of allergen exposition since early oral exposure to a potential food allergen is considered a tolerance inductor, whereas cutaneous exposure through an impaired skin barrier promotes sensitization [48]. From a pathogenic standpoint, a pivotal mechanism mediating gene–environment interactions is the induction of epigenetic changes by the environment such as DNA methylation and microRNA [49,50,51,52,53]. An elegant experimental study by Martino et al. conducted using integrated DNA methylation and transcriptomic profiling showed cumulative increases in epigenetic disruption at T cell activation genes and poorer lymphoproliferative responses in children who fail to resolve food allergy in later childhood as compared to infants resolving food allergy [54]. In turn, it has been observed that the developmental environment can lead to permanent changes in gene expression and DNA methylation [55]. MicroRNA’s possible role as a biomarker in several allergic diseases has recently gained attention. In particular, a research article by Yang et al. conducted on a food allergy mouse model suggested that miR-19a may be a target to regulate the immune tolerant status in the body [56].

Many risk factors have been identified or proposed in the development of food allergy [9]. Non-modifiable risk factors include sex (male sex in children), race/ethnicity, and genetic factors (e.g., polymorphisms in specific genes and HLA). Modifiable risk factors include obesity, diet, vitamin D deficiency, the hygiene hypothesis, the influence of the microbiome, and timing and the route of exposure to food. In physiological conditions, ingestion of harmless antigens, including food proteins, results in oral tolerance consisting of local and systemic immune non-response [57]. Specifically, oral tolerance occurs when a food antigen, having crossed the intestinal barrier, is processed by dendritic cells which present the derived food peptide to naïve T cells and induce their differentiation into antigen-specific regulatory T cells (T-regs). T-regs encourage the maintenance of tolerance through the expression of CTLA-4, which inhibits Th2 T cells, and the release of the cytokines TGF-β and IL-10. TGF-β and IL-10 suppress mast cells, eosinophils, and basophils (the effector cells that promote allergic symptoms), promote the keeping of IgA in the intestinal lumen and the production of IgG4, and reduce the production of IgE by B cells [58]. The impairment of oral tolerance mechanisms can trigger the development of FA [59].

The immune response implicated in FA can be IgE-mediated, non-IgE-mediated, or based on a mixed mechanism. IgE-mediated FA is the best clarified and can be divided into two phases: sensitization and elicitation [4]. Sensitization is defined as the condition in which food-specific IgE is detectable in the serum, becoming a possible trigger factor for clinical manifestations of FA. This phase occurs when a food allergen crosses a compromised epithelial barrier and is captured by dendritic cells in a context of inflammatory cytokines such as thymic stromal lymphopoietin (TSLP), IL-25, and IL-33, thereby inducing a Th2-type response [58,60,61,62]. Differentiated Th2 cells can migrate from draining lymph nodes into the intestinal lamina propria and secrete pro-inflammatory cytokines, such as IL-5 and IL-13, to further promote the differentiation of effector cells such as eosinophils and basophils [58,63,64]. In addition, innate lymphoid cells type 2 (ICL2) also play an important role in the onset of FA through the secretion of cytokines such as IL-4 and IL-13 [65]. IL-4 triggers B-cell class switching and the production of allergen-specific IgE that binds to FcεRI on the surface of mast cells and basophils. During the elicitation phase, when a new encounter with an allergen causes cross-linking of the IgE-FcεRI complexes, sensitized basophils and mast cells are activated and subsequently release mediators responsible for the classical symptoms of the immediate phase (type 1 hypersensitivity) [66] such as histamine, prostaglandins, leukotrienes, tryptase, and platelet-activating factor [58]. A late-phase reaction follows, due to the accumulation of inflammatory mediators produced by mast cells and basophils and to the activation of allergen-specific Th2 cells, which produce interleukins, promote eosinophilia, maintain allergen-specific IgE levels, and recruit additional inflammation cells causing tissue damage and perpetuation of inflammation [67,68].

How the allergic reaction to food allergens affects the various organs (e.g., gastrointestinal, respiratory, skin, and cardiovascular systems) depends on several factors. If cross-linking of the allergen with IgE-FcεRI complexes remains confined to resident mast cells close to the allergen entry site, degranulation and the release of vasoactive mediators determines local symptoms in the gastrointestinal tract (e.g., abdominal cramping, diarrhea) [69]. On the other hand, the local allergic reaction can trigger a sequence of possible multisystem inflammatory events with the involvement of the (i) respiratory tract (e.g., dyspnea, wheezing, stridor, hypoxemia); (ii) skin and/or mucous membranes (e.g., urticaria, itching, redness, angioedema); and (iii) cardiovascular system (e.g., hypotension, hypovolemia, arrhythmias, distributive or mixed shock multiorgan failure) [70]. The evolution from local to systemic symptoms could reflect differences in (i) the entity of antigen-induced mediator release in the gut and their autocrine, paracrine, and endocrine effects; (ii) the amount of antigen absorbed into the bloodstream; (iii) the distribution of allergen-activated mast cells; and (iv) differences in the amount of IgA and IgG antibodies that can neutralize the allergen before it can cross-link mast cell IgE-FcεRI complexes [69]. Local or systemic reactions induced by direct contact of the food allergens with skin or lung mast cells are also documented [71,72,73,74].

### 3.1. Different Route of Sensitization

The most obvious route of sensitization is through the transit of food in the intestine. However, exposure to allergens can also occur through the respiratory tract and the skin [75]. Sensitization to food allergens in the gastrointestinal tract leads to class 1 FA. The most important allergens included in class 1 FA are cow’s milk, chicken eggs, and legumes, and clinical manifestations often disappear during growth and are replaced by other manifestations of atopic syndrome in adulthood [76]. Class 2 FA develops because of respiratory sensitization to inhalant allergens. Examples of class 2 FA include food allergies to plant pollen [77,78]. In this type of FA, IgE antibodies to pollens recognize homologous epitopes on food proteins of plant origin [79]. Dermal exposure is another non-oral route of sensitization included in class 2 FA. A weakened skin barrier (i.e., the skin of children with eczema or atopic dermatitis) can allow the internalization of the antigen in the body and induce IgE synthesis [80]. This finding could explain why FA can develop before oral exposure to the suspected food, suggesting that sensitization to the food must somehow occur before ingestion. To explain this phenomenon, there are various hypotheses including that of a double exposure to allergens, which suggests that the risk of developing an FA depends on the timing, dose, route of exposure, and the balance between oral exposure (usually tolerogenic) and cutaneous or respiratory (usually allergenic) exposure [67]. Finally, exposure to allergens can also occur trans-placentally in the uterus. Intrauterine sensitization of milk, eggs, and peanuts has been reported [81].

### 3.2. Allergenic Properties of Food Allergens and Cross-Reactivity

Approximately 400 allergenic proteins from more than 170 foods can cause IgE-mediated allergic reactions [82]. Some proteins are specific to a single food, resulting in a specific FA. Other proteins can be present in different foods with small differences between them, configuring the picture of cross-reactive FA. Cross-reactivity is a phenomenon in which the immune system “mistakes” a new protein with an already known one [83]. This happens because of the similarity of one or more epitopes among different allergenic proteins. Therefore, a new allergen can trigger an antibody or cell-mediated response because of a previous sensitization to another allergen with a similar epitope(s) [6], which usually belongs to the same family [84]. For example, in the case of hazelnut allergy, sensitization can occur in two ways with two different clinical profiles. In the first case (food specific), the sensitization is defined as primary, and it is caused by highly stable proteins specifically present in hazelnuts [5,85]. In this case, the symptoms are usually severe and systemic. On the other hand, sensitization to hazelnuts can develop through cross-reactivity with (i) homologous proteins contained in pollens (pollen-food syndrome) [86] or (ii) homologous proteins contained in other plant-derived foods [79,87]. The clinical presentation is widely heterogeneous ranging from mild symptoms referred to as oral allergy syndrome (OAS) to severe reactions up to anaphylaxis [88]. Indeed, cross-reactive allergens in pollen and plant-derived foods include non-specific lipid transfer proteins (nsLTPs), profilins (e.g., Bet v 2), and pathogenesis-related class 10 (PR-10) (e.g., Bet v 1) [78]. Cross-reactive proteins from animal origins mainly include tropomyosins in invertebrate animals and parvalbumins in fish and amphibians [78].

## 4. Allergic Sensitization Profile in Different Geographical Areas

### 4.1. Allergic Sensitization Profile in Europe

The sensitization profile to various food allergens changes in different geographical areas [89]. In Europe, data show that the sensitization profile is very heterogeneous since some allergens are common to other Western countries (e.g., hazelnut, shellfish, cow’s milk, chicken egg…) whereas other plant-derived foods allergens are relevant in specific geographical areas [20]. In pediatric populations, the most common causes of FA are cow’s milk, chicken egg, hazelnuts, peanuts, apples, peaches, kiwis, and carrots with a high prevalence of hazelnuts, apples, peaches, kiwis, and carrots in northern and central European countries (pollen-related birch) and a high prevalence of foods of animal or other plant origin in Mediterranean areas and Iceland [23]. In the adult population, hazelnuts, peaches, apples, carrots, walnuts, melons, shellfish, sunflower seeds, and bananas are the foods most frequently associated with FA [20]. A reasonable explanation for this heterogeneity is found in pollen sensitization and cross-reactivity [7,87,90]. In fact, while FA in children occurs more frequently through primary sensitization (class 1 FA), in adults it occurs more frequently through cross-reactivity (class 2 FA) [20,23,91]. For example, in northern, central and eastern Europe, birch pollen is the most common [92] and cross-reactivity between specific IgEs against proteins related to Bet v 1 (i.e., the main birch pollen allergen) and homologous food allergens present in nuts, Rosaceae, and Apiaceae, can justify FA for hazelnut, apple, and peach which are the most frequent food allergens in these areas [20,93]. Peaches are a major cause of FA in Mediterranean areas where the high frequency of nsLTP sensitization can cause FA for nsLTP-related fruits [20,29]. Usually, nsLTPs cause an FA to fruit in the absence of pollen allergy; however, mugwort pollen nsLTP has been shown to cross-react with peach nsLTP and may be involved with the mugwort–peach allergy association frequently seen in some Mediterranean areas [94,95]. Again, the cross-reactivity of profilins present in grass and ragweed pollen can explain the high prevalence of FA for melon and wheat in some Mediterranean areas where grass pollen is abundant [20,87].

### 4.2. Allergic Sensitization Profile in Italy

In Italy, in adult populations, fruits and vegetables represent the most frequent cause of FA followed by shrimp, fish, milk, eggs, cereals, meat, snails, and Anisakis [29]. The most frequent route of sensitization is represented by cross-reactivity to a primary sensitizer and includes a large majority of patients with pollen–food allergy syndrome (including patients mono-sensitized to birch pollen Bet v 1 or sensitized to all seasonal airborne allergens and, hence possibly sensitized to profilin). The remaining patients have an FA related to primary sensitization to plant-derived foods (in particular, patients allergic to nsLTPs) or animal-derived foods. Across Italy, FA is more frequent in northern and central Italy than in southern Italy, but this difference seems to be totally due to the pollen–food allergy syndrome whose frequency progressively decreases southbound. No difference was found in the prevalence of allergy to fish, milk, eggs, snails, meat, cereals, and Anisakis. No significant difference was detected in the prevalence of FA for tree nuts, kiwi, legumes, and buckwheat between the different areas of the country; although, an allergy to pine nuts or buckwheat occurred more frequently in the north. Shrimp allergy is more frequent in the northern part of the country; on the contrary, fruit and vegetables are the most common sensitizing foods in southern Italy and nsLTPs were by far the most frequent allergen [29].

To confirm that fruit and vegetables were also the main sensitizers in our area, we analyzed the sensitization profile of 915 adult patients (aged over 14 years, 515 female, 400 male, average age 27.92 y) followed at the Allergology Clinic of Napoli Federico II Hospital from 2013 to 2022 for probable FA and tested for 22 food allergens by in vivo SPT and in vitro specific IgE determination (Table 1).

**Figure 1:**
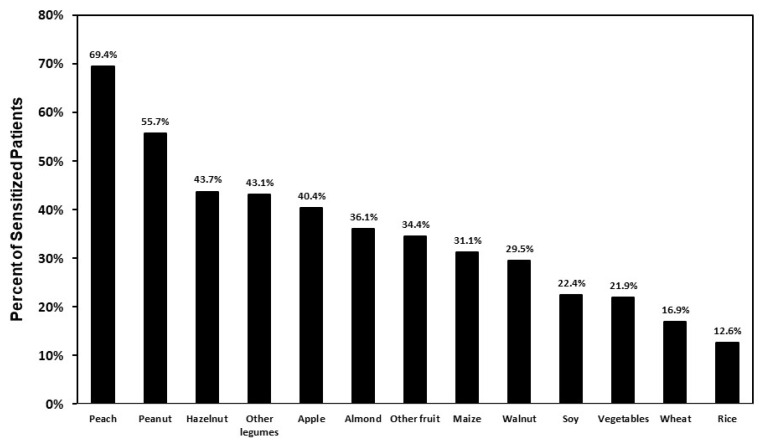
Percentage of sensitization of patients to all tested plant-derived foods (N = 915).

The percentage of sensitized patients to any plant-derived foods (fresh fruit 76.94%, legumes 65.03%, tree nuts 51.91%, cereals 38.80%, vegetables 21.86%) was consistently higher than those sensitized to any animal-derived origin foods (seafood 18.58%, cow’ milk proteins 7.65%, egg 4.92%). Among categories, Figure 1 shows the percentage of sensitization of patients to all tested plant-derived foods.

The most frequent allergen among plant-derived foods was peach (69.4%) followed by peanut (55.7%). The figure also shows that the percentage of sensitized patients to other plant-derived foods progressively decreased ranging from 43.7% of hazelnut to 12.6% of rice. These findings are in line with data from other Italian studies and confirm that peach is the main sensitizer in southern Italy [96,97]. It is well known that peach is a mixture of various molecular components that could extend the sensitization to other plant-derived foods through cross-reactive mechanisms [98,99,100,101,102,103].

### 4.3. Allergic Sensitization Profile in Other Countries

**Figure 2:**
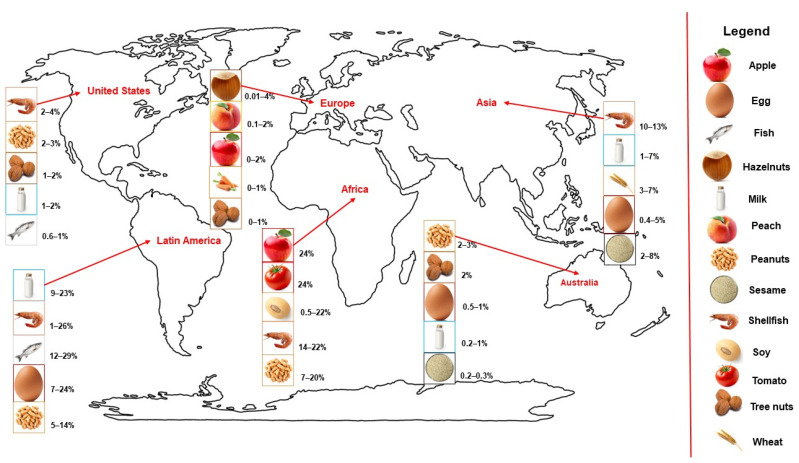
Most prevalent allergic sensitization in different geographical areas of the world.

In the United States, the most common allergens are shellfish, milk, peanuts, tree nuts, and fish in both adults and children [24,25]. In the Australian population, raw egg whites, peanuts, cow’s milk, sesame, and shellfish are the main allergens in children and are then replaced by peanuts and tree nuts in adolescence [26,27,28]. In Asia, shellfish allergy is the single most common FA among Asian populations, and cow’s milk and eggs are two of the most common food allergens in young children across Asia [30]. By contrast, some data suggest that the overall prevalence of FA for eggs, peanuts, and tree nuts in infants and schoolchildren is generally lower than in Western countries [19,30]. Additionally, wheat is emerging as an important cause of FA in Thailand, Korea, Japan, and Pakistan [19,30]. In Latin America, data on food sensitization profiles are scarce; however, the most common sensitizing foods reported include cow milk proteins, seafood, chicken eggs, and peanuts [19,30]. In Africa, the most frequently detected food allergen extracts were apple, tomato, soy, crab, and peanut [104]. Figure 2 summarizes the prevalence of the most common allergic sensitizations described in different geographical areas of the world. Wider oscillations in Africa and Latin America could be due to limited data on the food allergy prevalence in these countries. In addition, the data in different geographical areas are difficult to compare due to the different awareness of food allergy, the different methodologies used for diagnosis, and the data collection especially in developing countries.

## 5. Principal Molecular Components in Plant-Derived Food Allergy

### 5.1. Pru p 3 as the Prototype of Non-Specific Lipid Transfer Proteins (LTPs)

In the Mediterranean area, most allergic reactions to plant-derived food are due to sensitization to nsLTPs [105]. In particular, Pru p 3, the nsLTP from peach, is the primary sensitizer of LTP-mediated food allergies, involved in cross-reactive reactions with nsLTPs from apple, apricot, cherry, plum, raspberry, wheat, and others [84,106,107]. The common structural features of nsLTPs are the basis of their allergenic clinical cross-reactivity that is usually referred to as LTP syndrome [105,108]. nsLTPs are ubiquitous vegetable proteins involved in lipid membrane biosynthesis and act as pathogenesis-related proteins [109]. Together with the structurally closely related 2S-albumins and α-amylase/protease inhibitors, nsLTPs belong to the prolamin protein superfamily [109]. nsLTPs are found in various plant-derived foods [110,111] including fruits, nuts, and vegetables. Most of them are included in the list of 22 priority food allergens that we used for diagnostic purposes (Table 1): apple (Mal d 3), almond (Pru du 3), apricot (Pru ar 3), kiwi (Act d 10), strawberry (Fra a 3), cherry (Pru av 3), peanut (Ara h 9), hazelnut (Cor a 8), walnut (Jug r 3), bean (Pha v 3), pea (Pis s 3), lentil (Len c 3), tomatoes (Sola l 3), garlic (All a 3), carrot (Dau c 3), celery (Api g 2), maize (Zea m 14), wheat (Tri a 14), and rice (Ory s 14).

Two allergenic nsLTP subfamilies have been described: LTP1 of 9 kDa and LTP2 of 6–7 kDa [98,99]. These two families share the general molecular structure but show a rather low sequence similarity (about 30% identity) and differ in cysteine residues sited along the molecules [112]. The structural homology of nsLTP largely depends on the taxonomic relationships between the sources of origin and botanically related molecules. The molecules with a higher identity will have a greater likelihood of IgE cross-reactivity than taxonomically distant molecules [108]. Interestingly, unlike other cross-reactive molecules (e.g., profilins and PR-10), proportions of structural identity greater than 60% are rarely recorded, indicating that nsLTPs are homologous molecules with a low structural identity [113]. An important characteristic of nsLTPs is revealed by the 3D structure which is stabilized by four intramolecular disulfide bonds that confer high resistance to proteolytic digestion and heat treatment. As a consequence, allergic reactions to nsLTPs are usually severe and systemic [111]. However, it should be noted that in some cases, sensitized individuals show mild symptoms or remain asymptomatic [98]. Although nsLTP sensitization appears to be most prevalent in southern Europe, it is also emerging in European countries other than the Mediterranean areas including Austria, France, Belgium, Germany, Poland, and the United Kingdom. Outside Europe, sensitization to nsLTPs appears to be clinically relevant in some areas of China while it has not yet been reported in the Americas or Africa [99]. It should be noted that in areas other than the Mediterranean, the nsLTP acting as primary sensitizer is usually different from Pru p 3 [114,115,116,117].

### 5.2. Other Molecular Components in Peach

In peach, besides Pru p 3, at least three other molecular components are known and characterized as follows: Pru p 1, Pru p 4, and Pru p 7. Pru p 1 belongs to the pathogenesis-related protein family 10 (PR-10) [118], which comprise a unique class of phytoproteins highly conserved in many species of plants. PR-10s are mainly cytosolic proteins, constitutively expressed in different plant tissues (e.g., roots, flowering compartments, fruits, and pollen grains). Their expression is upregulated in conditions of biotic stress (e.g., viral, bacterial, or fungal infections), or chemical and physical stress (e.g., cold, salinity, drought, oxidative stress, ultraviolet radiation, and wounds) [119]. Therefore, it is possible that PR-10 proteins are implicated in plant defense mechanisms [120]. PR-10 proteins have a molecular weight of approximately 17 kDa [121]. Bet v 1, the major birch pollen allergen, is the prototype of the PR-10 family. Other members of this family are present in plant-derived foods such as Rosaceae (e.g., Pru p 1 in peach, Mal d 1 in apple, Pru a 1 in cherry, Pyr c 1 in pear), Apiaceae (e.g., Api g 1 in celery, Dau c 1 in carrot), soybean (Gly m 4), mung bean (Vig r 1), hazelnut (Cor a 1), and peanut (Ara h 8) [122]. These proteins share a high degree of amino acid sequence similarity with Bet v 1 resulting in a similar tertiary structure [123]. Thus, Bet v 1-specific IgE antibodies cross-react with these food proteins very frequently determining immediate allergic reactions upon consumption of the respective foods [124]. However, PR-10 proteins are heat labile and unstable to pepsin digestion [125]. As a consequence, the symptoms are usually mild and characterized by oral itching or burning [126].

Pru p 4 belongs to the family of profilins which are the most widespread allergens in the plant kingdom. Profilins are cytosolic proteins present in all eukaryotic cells and play a structural role in the regulation of the polymerization of actin filaments [122]. Profilins have a molecular mass of 12–15 kDa, and a highly conserved structure with 70–85% of homology among different species. They were first identified as a minor allergen in birch pollen and named Bet v 2 [113]. Afterward, they have been identified in various foods such as peach (Pru p 4), peanuts (Ara h 5), soybean (Gly m 3), and celery (Api g 4) [111,127]. Similarly to PR-10, profilins are quite sensitive to heat denaturation and gastric digestion, and thus FA caused by these proteins is usually confined to the OAS elicited by raw foodstuffs [122]. Pru p 7 (peamaclein) is a gibberellin-regulated protein (GRP) identified and registered as a peach allergen only in 2013 [128]. The existence of a new food allergen was suspected with the observation of peach-induced systemic allergic reactions in patients who did not show IgE reactivity for Pru p 1, Pru p 3, and Pru p 4 [129]. GRPs are small basic proteins, implicated in the defense of plants from biotic and abiotic agents, with a molecular weight of 7 kDa and a structure characterized by 12 cysteines and 6 disulfide bridges, which confer resistance to proteolytic digestion and heat treatment [130]. As a consequence, Pru p7 is associated with severe allergic reactions elicited by peach with the severity correlated with the concentration of IgE [131]. Sensitization to Pru p 7 is more prevalent in the areas with high exposure to cypress pollen that acts as a primary sensitizer. In fact, a proportion of individuals sensitized to cypress shows cross-reactivity with Pru p 7 [131]. To date, cross-reactivity of Pru p 7 with other GRPs has been demonstrated with Pru m 7 (Japanese apricot), Cit s 7 (orange), and Pun g 7 (pomegranate) [128,132,133,134,135,136].

## 6. Spectrum of Clinical Severity in Food Allergy

Food allergies can manifest with various clinical symptoms, from mild to severe, up to anaphylaxis [137]. Skin and mucous membrane-related symptoms are common in food allergies and can include urticaria, eczema, itchy or tingling sensation in the mouth or the throat, and angioedema [138,139,140,141]. Food allergies can also affect the gastrointestinal tract, and the respiratory and cardiovascular systems leading, in severe cases, to a systemic reaction and finally to anaphylaxis [82,142]. A standardized classification of FA severity is currently lacking, and the World Allergy Organization (WAO) has started a project to develop an international system for defining and classifying the severity associated with food allergy (“DEfinition of Food Allergy SEverity”, DEFASE) [143]. However, it is currently accepted that symptoms affecting the airways, cardiovascular system, and/or consciousness constitute a severe reaction [144].

The symptoms experienced during an adverse reaction can vary between individuals depending on a multitude of factors: (i) some are related to the host, for example, the age (teen age and adult youth represent a risk factor for fatal reactions), allergic comorbidities (e.g., asthma and mastocytosis), and previous adverse reactions; (ii) some are related to the allergen (e.g., dose of allergen and food processing); (iii) some are related to the IgE-mediated immune response (e.g., IgE level); and (iv) others are probably unknown [145]. Although it would be highly desirable to recognize prognostic risk factors, the severity of food allergic reactions remains largely unpredictable [146]. Several attempts have been made to analyze the relationship between the dose/level of allergen exposure and the severity of the adverse reaction. However, data are complex and inconclusive since most datasets have shown that severe reactions can occur at all levels of allergen exposure [142]. For sure, the allergenicity of food proteins is influenced by the 3D structure of food allergens that confers resistance to heat and gastric digestion [147]. For example, the presence of IgE in molecular components more resistant to modification through food processing can help identify individuals with more severe reactions [148]. This happens for nsLTPs (which are heat-stable, resistant to proteolytic digestion, and usually associated with severe adverse reactions) [149] in contrast to PR-10 proteins and profilins (which are heat labile, unstable to pepsin digestion, and usually associated with mild FA symptoms) [122,126]. However, these indications do not represent a rule.

Again, the potential use of specific IgE levels to predict the severity of allergic reactions is conflicting and it seems that high levels of IgE sensitization (wheal SPT and/or food allergen-specific IgE) are usually associated with clinical reactivity but do not predict the occurrence and the severity of the reaction or the onset of anaphylaxis [142,145]. For Pru p 3, it has been reported that high levels of specific IgE are associated with systemic reactions to peach [98,142]. However, co-sensitization to Pru p 3 and Pru p 1, Pru p 4, or both, appears to play a protective role, resulting in a lower frequency of severe systemic reactions [98,150]. For this reason, CRD analysis for molecular components can be useful in predicting a higher or lower risk of anaphylaxis depending on the sensitization profile. However, though an association between nsLTP sensitization and severity of FA symptoms has been reported [151], nsLTP sensitization can show extremely variable clinical pictures, ranging from contact urticaria, oral allergy syndrome, food-dependent exercise-induced anaphylaxis, urticaria/angioedema, and anaphylaxis. Sometimes sensitization is discovered accidentally during the diagnostic work-up carried out for various reasons [152]. Thus, the heterogeneity of symptoms is a common characteristic in nsLTP-related FA and depends on various factors. First the concentration of nsLTPs in the various foods depends on the variety of fruit, its maturity, and preservation methods [153]. Cross-reactivity among nsLTPs, determined by structural similarity [84], interferes with the heterogeneity of clinical manifestations associated with LTP-related FA. The risk of cross-reactivity is more complex than only estimating frequency homology. Indeed, cross-reactivity can also occur for foods distant from a taxonomic point of view. For example, patients with FA for peach cross-react more frequently with walnuts than pears, despite the sequence homology between the LTP of the latter and Pru p3 being decidedly highest [154]. Therefore, the cross-reactivity between nsLTPs could also be influenced by the tertiary structure, the matrix effect, the percentage of LTPs, and the presence of linear or conformational epitopes [136]. From previous studies, it has been demonstrated that the level of Pru p 3 sIgE is fundamental for the occurrence of cross-reactivity with botanically related (Rosaceae) and unrelated plant foods. However, no cut-off levels have been established for identifying patients with clinically significant allergy [98].

The heterogeneity of symptoms, together with the unpredictability of their evolution and the absence of predictive factors of severity of the allergic reactions have important clinical implications and make the management of food allergy very challenging. In fact, both in the diagnostic phase and in the management and therapy, all the anamnestic elements and diagnostic tests should be integrated to make decisions that can radically change the patients’ eating habits and lifestyles. The starting point for diagnosing FA, is a careful collection of the clinical history, of clinical manifestations, and eventual suspected association with food, keeping in mind possible disorders or symptoms which could be mistaken for allergic reactions to food. Additional diagnostic information is obtained by appropriately selecting and interpreting tests, such as SPTs, sIgE measurements, and OFCs. CRD provides a major step in improving the accuracy of diagnosing IgE-mediated sensitizations in FA, identifying the specific sensitization or possible cross-reactivity between allergens. All the information collected helps to define the patient’s allergic profile, to evaluate the risk of severe reactions, and to make therapeutic decisions [46,82,155,156]. Given the absence of a cure, effective management of food allergy requires avoiding ingestion of culprit food (or in some cases avoidance of raw foods). The correct choice of foods to be excluded is essential because it will strongly impact the patient’s quality of life [157]. Obtaining effective avoidance can be complex and requires careful education of the patient, his family, and eventual caregivers, and their compliance [46,158]. Nutritional counseling could be necessary because allergen avoidance diets can cause nutritional deficiencies and therefore growth monitoring for children with FA is highly recommended [46]. On the other hand, dietary restrictions can have a significant psychological impact on patients, leading to depression, psychological distress, and eating disorders [159,160,161]. Indeed, it is not possible to obtain a total suppression of the risk of adverse reactions to foods because of various factors (i.e., poor patient compliance in avoiding unsafe foods, onset of allergic reaction to previously tolerated foods, risk of casual ingestion of offending foods). For these reasons, self-injectable epinephrine remains the cornerstone of therapy for the prompt treatment of severe allergic reactions [162].

## 7. Conclusions

The epidemiology of FA shows a high variability worldwide in terms of prevalence, sensitization profile, and clinical expression. To explain this heterogeneity, there are several hypotheses including different allergen exposure related to the environmental setting and eating habits and route of sensitization (gastrointestinal vs. respiratory tract and the skin) [67]. Another reasonable explanation for this heterogeneity is found in the cross-reactivity between homologous proteins or common epitopes shared by food and inhaled allergens. In southern Italy, Pru p 3, the peach nsLTP, is the primary sensitizer acting as a driver for other nsLTP sensitization. Due to its high cross-reactivity it can create complex profiles of sensitizations with heterogeneous clinical manifestations ranging from mild to severe reactions up to anaphylaxis. Considering the potential life-threatening features of nsLTPs-related FA, proper management taking into account the patient’s geographical setting is highly recommended to build a personalized approach for managing FA across the world in the precision medicine era.

## Figures and Tables

**Table 1:** Percentage of sensitization to food allergens in our cohort of patients with food allergy (N = 915). Methods: SPTs were performed in accordance with the EAACI guidelines, testing the following extracts (Roxall Italia SRL; Rome, Italy; Lofarma SpA, Milan, Italy): egg (egg white and yolk), cow’s milk (α-lactalbumin, β-lactoglobulin, casein), seafood (cod for fish, shrimp for shellfish, mussel for mollusk), cereals (wheat, maize, rice), tree nuts (almond, walnut, hazelnut), legumes (peanut, soy, and one other legume among bean, pea, or lentil), fresh fruit (peach, apple, and one other fruit among apricot, kiwi, strawberry, or cherry), vegetables (two among tomatoes, garlic, onion, carrot, or celery), a negative control (glycerinated saline), and a positive control (histamine). A skin prick test response was considered positive if the wheal diameter was 3 mm greater than that of the glycerinated saline control. The dosage of specific IgE in serum was quantized using the ImmunoCap assay method and considered positive for a value > 0.50 KU/L.

Food	Percentage of Positive Patients (%)
**Egg**	**4.92%**
Egg white	3.83%
Yolk	3.28%
**Cow’s Milk**	**7.65%**
α-lactalbumin	3.93%
β-lactoglobulin	5.68%
Casein	4.81%
**Sea Food**	**18.58%**
Cod (fish)	8.20%
Shrimp (shellfish)	10.93%
Mussel (mollusk)	5.90%
**Cereals**	**38.80%**
Wheat	16.94%
Maize	31.15%
Rice	12.57%
**Tree Nuts**	**51.91%**
Almond	36.07%
Walnut	29.51%
Hazelnut	43.72%
**Legumes**	**65.03%**
Peanut	55.74%
Soy	22.40%
Other legumes (bean, pea, or lentil)	43.17%
**Fresh Fruit**	**76.94%**
Peach	69.40%
Apple	40.44%
Other fruit (apricot, kiwi, strawberry, or cherry)	34.43%
**Vegetables (tomatoes, garlic, onion, carrot, or celery)**	**21.86%**

## Data Availability

Not applicable.
